# Supplementary material for: A community-developed extension to Darwin Core for reporting the chronometric age of specimens
Source: PLoS One. 2022 Sep 15;17(9):e0261044. doi: 10.1371/journal.pone.0261044 (PMC9477364; doi:10.1371/journal.pone.0261044)
Supplement: S1 Table — (DOCX) [file pone.0261044.s001.docx]

**SUPPLEMENTAL MATERIALS**

Table S1. Counts for the number of records with reporting in each Darwin Core GeologicalContext field in our sample dataset of 85,000 FossilSpecimen occurrence records from VertNet.

| **Darwin Core GeologicalContext term** | **Number of Records With a Value Reported** |
| --- | --- |
| earliestEonOrLowestEonothem | 4,930 |
| latestEonOrHighestEonothem | 4,930 |
| earliestEraOrLowestErathem | 21,755 |
| latestEraOrHighestErathem | 7,507 |
| earliestPeriodOrLowestSystem | 24,182 |
| latestPeriodOrHighestSystem | 9,185 |
| earliestEpochOrLowestSeries | 25,242 |
| latestEpochorHighestSeries | 9,150 |
| earliestAgeOrLowestStage | 8,944 |
| latestAgeOrHighestStage | 2,571 |
| lowestBiostratigraphicZone | 15,397 |
| highestBiostratigraphicZone | 6,151 |
| lithostratigraphicTerms | 4,473 |
| group | 8,015 |
| formation | 19,989 |
| member | 3,958 |
| bed | 1,479 |
| Total records that have at least one geologicalContext field with a value | 32,813 |
