# Supplementary material for: A community-developed extension to Darwin Core for reporting the chronometric age of specimens
Source: PLoS One. 2022 Sep 15;17(9):e0261044. doi: 10.1371/journal.pone.0261044 (PMC9477364; doi:10.1371/journal.pone.0261044)
Supplement: S2 Table — (DOCX) [file pone.0261044.s002.docx]

Table S2. Chronometric Age Extension example for a specimen from the North Midden archaeological site in Florida.

| Field name | Value |
| --- | --- |
| occurrenceID | ff2ada6a-12f5-4f51-ac09-c31b45bfced4 |
| chronometricAgeProtocol | AMS |
| verbatimChronometricAge | 3020-2610 cal BC, intercept 2860 cal BC |
| earliestChronometricAge | 3020 |
| earliestChronometricAgeReferenceSystem | cal BC |
| latestChronometricAge | 2610 |
| latestChronometricAgeReferenceSystem | cal BC |
| materialDated | *Crassostrea virginica* right valve specimen from Feature 17 |
| materialDatedID | fb437449-6d2c-459f-8f76-2d531d0e8cc8; 702b306d-f167-44d0-a5c9-890ece2b8783 |
| materialDatedRelationship | shell within Feature 17 |
| chronometricAgeReferences | 2006 Archaeological Data Recovery and Mitigation at The North Midden Site (8FL216) Flagler County, Florida By Ryan O. Sipe, Greg S. Hendryx, and Neill J. Wallis; ESI Report of Investigations No. 980; Report submitted to Hammock Beach River Club, LLC; Report on file with Division of Historical Resources, Florida Department of State, Tallahassee, Florida, U.S.A. |
| chronometricAgeRemarks | One of the *Crassostrea virginica* right valve specimens from North Midden Feature 17 was chosen for AMS dating, but it is unclear exactly which specimen it was. |
| chronometricAgeDeterminedBy | Environmental Services Inc., Beta Analytic Inc. |
| chronometricAgeDeterminedDate | 2006 |
