# Supplementary material for: A community-developed extension to Darwin Core for reporting the chronometric age of specimens
Source: PLoS One. 2022 Sep 15;17(9):e0261044. doi: 10.1371/journal.pone.0261044 (PMC9477364; doi:10.1371/journal.pone.0261044)
Supplement: S3 Table — (DOCX) [file pone.0261044.s003.docx]

Table S3. North Midden example as it appears in a VertNet record with JSON in dwc:dynamicProperties expressing the Chronometric Age Extension (and other archaeological provenience information as described in LeFebvre et al., 2019) so it is more easily viewable by users.

| Field name | Value |
| --- | --- |
| occurrenceID | ff2ada6a-12f5-4f51-ac09-c31b45bfced4 |
| dynamicProperties | {"Site Number":"8FL216","Provenience":{"Block":"C","Unit":"15","Level":"10","Feature":"17"},"Sum weight in grams of all elements in catalog number":"1.03","ChronometricAges": [{"chronometricAgeID":"", {"verbatimChronometricAge":"3020-2610 cal BC, intercept 2860 cal BC","chronometricAgeProtocol":"AMS","uncalibratedChronometricAge":"","chronometricAgeConversionProtocol":"","earliestChronometricAge":"3020","earliestChronometricAgeReferenceSystem":"cal BC","latestChronometricAge":"2610","latestChronometricAgeReferenceSystem":"cal BC","chronometricAgeUncertaintyInYears":"","chronometricAgeUncertaintyMethod":"","materialDated":"Crassostrea virginica right valve specimen from Feature 17","materialDatedID":"fb437449-6d2c-459f-8f76-2d531d0e8cc8; 702b306d-f167-44d0-a5c9-890ece2b8783","materialDatedRelationship":"shell within Feature 17","chronometricAgeDeterminedBy":"Environmental Services Inc., Beta Analytic Inc.","chronometricAgeDeterminedDate":"2006","chronometricAgeReferences":"2006 Archaeological Data Recovery and Mitigation at The North Midden Site (8FL216) Flagler County, Florida By Ryan O. Sipe, Greg S. Hendryx, and Neill J. Wallis; ESI Report of Investigations No. 980; Report submitted to Hammock Beach River Club, LLC; Repo","chronometricAgeRemarks":"One of the Crassostrea virginica right valve specimens from North Midden Feature 17 was chosen for AMS dating, but it is unclear exactly which specimen it was."}] } |
