# Supplementary material for: A community-developed extension to Darwin Core for reporting the chronometric age of specimens
Source: PLoS One. 2022 Sep 15;17(9):e0261044. doi: 10.1371/journal.pone.0261044 (PMC9477364; doi:10.1371/journal.pone.0261044)
Supplement: S4 Table — (DOCX) [file pone.0261044.s004.docx]

Table S4. Chronometric Age Extension example for a specimen from the Baptizing Springs archaeological site in Florida.

| Field name | Value |
| --- | --- |
| occurrenceID | 1148749e-ae0e-4c3b-b517-885bc8e56d88 |
| chronometricAgeProtocol | comparison between site artifacts with known date ranges and ethnohistoric records referencing the region and cultural context of the study |
| materialDatedRelationship | correlations between artifact types identified at the site and ethnohistoric accounts of missions from the region |
| verbatimChronometricAge | approximately 1620 to approximately 1656 |
| earliestChronometricAge | 1620 |
| earliestChronometricAgeReferenceSystem | AD |
| latestChronometricAge | 1656 |
| latestChronometricAgeReferenceSystem | AD |
| chronometricAgeReferences | Loucks, L.J. (1979) Political and Economic Interactions between Spaniards and Indians: Ethnohistorical and Archaeological Perspectives of the Mission System in Florida. Ph.D. dissertation, University of Florida, Gainesville. Loucks, J.L. (1991) Spanish-Indian Interaction on the Florida Missions: The Archaeology of Baptizing Spring. Florida Anthropologist 44(2-4):204-213. Loucks, L.J. (1993) Spanish-Indian Interaction on the Florida Missions: The Archaeology of Baptizing Spring. In The Spanish Missions of La Florida (B.G. McEwan, ed.) 193-216. Gainesville: University Press of Florida. |
| chronometricAgeDeterminedBy | Jill Loucks |
| chronometricAgeDeterminedDate | 1991 |
