# Supplementary material for: A community-developed extension to Darwin Core for reporting the chronometric age of specimens
Source: PLoS One. 2022 Sep 15;17(9):e0261044. doi: 10.1371/journal.pone.0261044 (PMC9477364; doi:10.1371/journal.pone.0261044)
Supplement: S5 Table — (DOCX) [file pone.0261044.s005.docx]

Table S5. Baptizing Springs example as it appears in a VertNet record with JSON in dwc:dynamicProperties expressing the Chronometric Age information so it is more easily viewable by users.

| Field name | Value |
| --- | --- |
| occurrenceID | 1148749e-ae0e-4c3b-b517-885bc8e56d88 |
| dynamicProperties | {"Site Number":"8SU65","Provenience":{"Field Specimen Number":"55","Unit":"542N 497E","Zone":"I"},"Sum weight in grams of all elements in catalog number for taxon":" 18.17","ChronometricAges": [{"chronometricAgeID":"", {"verbatimChronometricAge":"approximately 1620 to approximately 1656","chronometricAgeProtocol":"comparison between site artifacts with known date ranges and ethnohistoric records referencing the region and cultural context of the study","uncalibratedChronometricAge":"","chronometricAgeConversionProtocol":"","earliestChronometricAge":"1620","earliestChronometricAgeReferenceSystem":"AD","latestChronometricAge":"1656","latestChronometricAgeReferenceSystem":"AD","chronometricAgeUncertaintyInYears":"","chronometricAgeUncertaintyMethod":"","materialDated":"","materialDatedID":"","materialDatedRelationship":"correlations between artifact types identified at the site and ethnohistoric accounts of missions from the region","chronometricAgeDeterminedBy":"Jill Loucks","chronometricAgeDeterminedDate":"1991","chronometricAgeReferences":"Loucks, L.J. (1979) Political and Economic Interactions between Spaniards and Indians: Ethnohistorical and Archaeological Perspectives of the Mission System in Florida. Ph.D. dissertation, University of Florida, Gainesville. Loucks, J.L. (1991) Spanish-In","chronometricAgeRemarks":""}] } |
