# Supplementary material for: A community-developed extension to Darwin Core for reporting the chronometric age of specimens
Source: PLoS One. 2022 Sep 15;17(9):e0261044. doi: 10.1371/journal.pone.0261044 (PMC9477364; doi:10.1371/journal.pone.0261044)
Supplement: S6 Table — (DOCX) [file pone.0261044.s006.docx]

Table S6. Chronometric Age Extension example (record one of two) for a specimen from the Hawk Rim paleontological site in Oregon.

| Field name | Value |
| --- | --- |
| occurrenceID | bea3adb1-dca4-478d-8a4d-29c0c95d9887 |
| chronometricAgeProtocol | specimen date constrained by in situ position between the Hawk Rim Tuff (dated with U-Pb) and Double Tuff (dated with Ar-ar) |
| earliestChronometricAge | 16.44 |
| earliestChronometricAgeReferenceSystem | Ma |
| chronometricAgeUncertaintyInYears | 16000 |
| materialDated | Hawk Rim Tuff |
| chronometricAgeRemarks | Win N. F. McLaughlin, Samantha S. B. Hopkins & Mark D. Schmitz (2016) A new late Hemingfordian vertebrate fauna from Hawk Rim, Oregon, with implications for biostratigraphy and geochronology, Journal of Vertebrate Paleontology, 36:5, DOI:10.1080/02724634.2016.1201095 |
