# Supplementary material for: A community-developed extension to Darwin Core for reporting the chronometric age of specimens
Source: PLoS One. 2022 Sep 15;17(9):e0261044. doi: 10.1371/journal.pone.0261044 (PMC9477364; doi:10.1371/journal.pone.0261044)
Supplement: S8 Table — (DOCX) [file pone.0261044.s008.docx]

Table S8. Hawk Rim example as it appears in a record with JSON in dwc:dynamicProperties expressing the Chronometric Age information so it is more easily viewable by users.

| Field name | Value |
| --- | --- |
| occurrenceID | bea3adb1-dca4-478d-8a4d-29c0c95d9887 |
| dynamicProperties | {"ChronometricAges": [{"chronometricAgeID":"", {"verbatimChronometricAge":"","chronometricAgeProtocol":"specimen date constrained by in situ position between the Hawk Rim Tuff (dated with U-Pb) and Double Tuff (dated with Ar-ar)","uncalibratedChronometricAge":"","chronometricAgeConversionProtocol":"","earliestChronometricAge":"","earliestChronometricAgeReferenceSystem":"","latestChronometricAge":"16.26","latestChronometricAgeReferenceSystem":"Ma","chronometricAgeUncertaintyInYears":"16000","chronometricAgeUncertaintyMethod":"","materialDated":"Hawk Rim Tuff","materialDatedID":"","materialDatedRelationship":"","chronometricAgeDeterminedBy":"","chronometricAgeDeterminedDate":"","chronometricAgeReferences":"Win N. F. McLaughlin, Samantha S. B. Hopkins & Mark D. Schmitz (2016) A new late Hemingfordian vertebrate fauna from Hawk Rim, Oregon, with implications for biostratigraphy and geochronology,Journal of Vertebrate Paleontology, 36:5, DOI:10.1080/02724634.2","chronometricAgeRemarks":""}, {"chronometricAgeID":"", {"verbatimChronometricAge":"","chronometricAgeProtocol":"specimen date constrained by in situ position between the Hawk Rim Tuff (dated with U-Pb) and Double Tuff (dated with Ar-ar)","uncalibratedChronometricAge":"","chronometricAgeConversionProtocol":"","earliestChronometricAge":"16.44","earliestChronometricAgeReferenceSystem":"Ma","latestChronometricAge":"","latestChronometricAgeReferenceSystem":"","chronometricAgeUncertaintyInYears":"50000","chronometricAgeUncertaintyMethod":"","materialDated":"Double Tuff","materialDatedID":"","materialDatedRelationship":"","chronometricAgeDeterminedBy":"","chronometricAgeDeterminedDate":"","chronometricAgeReferences":"Win N. F. McLaughlin, Samantha S. B. Hopkins & Mark D. Schmitz (2016) A new late Hemingfordian vertebrate fauna from Hawk Rim, Oregon, with implications for biostratigraphy and geochronology,Journal of Vertebrate Paleontology, 36:5, DOI:10.1080/02724634.2","chronometricAgeRemarks":""}] } |
